# Decoding the Spectrum of Anorexia Nervosa: Clinical Impact, Molecular Insights, and Therapeutic Perspectives

**DOI:** 10.3390/biom15111559

**Published:** 2025-11-06

**Authors:** Dimitris C. Kounatidis, Natalia G. Vallianou

**Affiliations:** 1 Diabetes Center, First Department of Propaedeutic Internal Medicine, Medical School, National and Kapodistrian University of Athens, Laiko General Hospital, 11527 Athens, Greece; dimitriskounatidis82@outlook.com; 2 First Department of Internal Medicine, Sismanogleio General Hospital, 15126 Athens, Greece

**Keywords:** anorexia nervosa, autophagy, gut–brain axis, hormones, immunity, neuromodulation, novel therapies, oxidative stress, systemic complications

## Abstract

Anorexia nervosa (AN) is a severe psychiatric disorder with the highest mortality rate among mental illnesses, characterized by an intense fear of weight gain, persistent restriction of energy intake, and a distorted perception of body image. Despite decades of investigation, the pathogenesis of AN is only partially understood and is recognized as multifactorial, involving genetic, sociocultural, and neurobiological determinants. Beyond its core psychopathological features, AN leads to a wide spectrum of systemic complications, including cardiovascular, renal, skeletal, and endocrine dysfunctions. Increasing evidence implicates autophagy and oxidative stress as key molecular mechanisms underpinning its pathophysiology, while growing attention has been directed toward immune dysregulation and alterations in the gut–brain axis as potential mediators of disease onset and progression. Therapeutic advances, however, remain limited. Current management relies primarily on nutritional rehabilitation and psychotherapeutic interventions, while treatment outcomes are constrained by high relapse rates and the lack of pharmacological agents with proven efficacy. In this context, a more comprehensive understanding of the clinical spectrum and molecular substrates of AN is essential to improving prognosis and guiding the development of novel therapeutic strategies. This narrative review synthesizes current evidence on the non-psychopathological dimensions of AN, encompassing its clinical manifestations, systemic complications, and implicated molecular pathways. It also appraises existing treatment modalities and examines emerging interventions with translational potential. Overall, this review aims to provide clinicians and researchers with an updated and integrative overview of AN, shedding light on novel directions in ongoing research.

## 1. Introduction

Anorexia nervosa (AN) is a complex and potentially life-threatening eating disorder (ED) defined by persistent restriction of energy intake, intense fear of weight gain, and a distorted body image, resulting in clinically significant underweight and widespread systemic consequences. AN carries the highest mortality rate among psychiatric illnesses, primarily due to the medical sequelae of malnutrition. Mortality risk is further increased in the presence of psychiatric comorbidities, particularly within the first decade after diagnosis, with suicide representing a leading cause of death [1,2]. The disorder disproportionately affects females, with a lifetime prevalence of up to 4%, compared with 0.3% in males. While overall incidence has remained relatively stable, diagnoses among individuals younger than 15 years are rising, likely due to earlier onset and improved detection [3].

According to the Diagnostic and Statistical Manual of Mental Disorders, Fifth Edition (DSM-5), AN is defined by three cardinal features: (i) restriction of energy intake resulting in significantly low body weight relative to age, sex, developmental stage, and health status; (ii) intense fear of weight gain or persistent behaviors that prevent weight restoration; and (iii) disturbance in body image, excessive influence of body weight or shape on self-evaluation, or failure to recognize the severity of underweight [4]. Two primary subtypes of AN are delineated, the restricting type and the binge-eating/purging type, distinguished by the presence of recurrent binge eating or purging behaviors [4,5]. The DSM-5 further recognizes atypical AN, in which the core psychopathological features are present despite the individual maintaining a weight within or above the normal range, potentially exhibiting distinct patterns in the frequency and severity of medical complications [4,6].

AN pathogenesis remains only partially understood, yet converging evidence supports a multifactorial etiology encompassing genetic, epigenetic, sociocultural, psychiatric, and biological influences. Recent molecular studies have implicated the *WDR6* gene in disease susceptibility, and differential DNA methylation patterns have also been observed in women with AN compared with both healthy controls and remitted cases. These motifs are particularly notable in genes such as *SYNJ2*, *PRKAG2*, and *STAT3*, which are involved in psychiatric, metabolic, and immune pathways [7,8]. Emerging research data also highlight autophagy, oxidative stress, immune dysregulation, and alterations in the gut microbiota as pivotal mechanisms contributing to disease onset and progression [9,10].

Treatment of AN remains particularly challenging due to patients’ frequent resistance to weight restoration and the uncertainty often reported by non-specialist physicians managing complex inpatient cases. These barriers underscore the necessity of multidisciplinary care, specialist involvement, and targeted physician training to enhance clinical confidence and decision-making [11,12]. Integrating advances in the molecular and systemic understanding of AN with its clinical manifestations is essential for optimizing current management and informing the development of novel therapeutic strategies.

This narrative review provides a comprehensive synthesis of current knowledge on the clinical spectrum of AN, highlighting its systemic repercussions and associated laboratory correlates. It also incorporates emerging insights into the disorder’s molecular mechanisms, including the roles of the immune system and gut microbiome, while examining contemporary therapeutic strategies. Finally, the review identifies key areas for future research aimed at improving treatment outcomes.

## 2. Literature Search

For the purposes of this narrative review, a comprehensive literature search was conducted in the PubMed (NIH) database using the keywords “anorexia nervosa”, “pathogenesis”, and “treatment”. The search primarily focused on publications from the past five years, yielding a total of 441 records. Priority was given to research articles, systematic and narrative reviews, clinical trials, and meta-analyses relevant to the pathophysiology and therapeutic approaches to AN. Reference lists of included papers were further screened to identify additional pertinent studies. Given the breadth of the literature retrieved, it is acknowledged that not all studies could be discussed in exhaustive detail within the scope of the present review.

## 3. Neurological Manifestations in Anorexia Nervosa

In AN, central nervous system (CNS) alterations are characterized primarily by atrophy of both gray and white matter, often accompanied by deficits in higher-order cognitive functions. Neuroimaging studies have revealed ventricular enlargement and widening of cortical sulci, indicative of diffuse cerebral volume loss [13,14,15,16,17]. A recent study by Gupta et al. demonstrated that even in early-stage, acute restrictive-type AN, structural brain changes resembled those typically associated with accelerated aging. Using magnetic resonance imaging (MRI) coupled with machine learning–based brain-age analysis, this study identified marked deviations from normative brain-age profiles. Partial reversal of these alterations was observed following nutritional rehabilitation, although complete restoration was not achieved in all cases [18].

Neuropsychological investigations have identified cognitive rigidity as a common feature in AN, persisting in some individuals despite weight restoration [19]. Morphometric analyses have localized volume reductions to regions such as the thalamus, hippocampus, and cerebellum, areas integral to sensory integration, memory, and motor coordination. At the cellular level, chronic malnutrition deprives neural tissue of essential substrates, including polyunsaturated fatty acids (PUFAs), potentially impairing membrane integrity, synaptic transmission, and myelination, thereby contributing to the observed structural deficits [20,21,22,23,24,25]. Notably, individuals with AN, particularly those experiencing rapid weight loss, are at risk for Wernicke’s encephalopathy due to severe thiamine malnourishment [26].

Evidence also indicates that women with AN exhibit diminished sympathetic nervous system activity, likely reflecting an adaptive response to chronic energy restriction [27]. Peripheral nervous system (PNS) involvement has further been reported, manifesting as distal weakness, paresthesia, or, in severe cases, foot drop. These deficits are thought to result from direct axonal injury secondary to nutrient depletion, compounded by connective tissue alterations that compromise neural support structures. Most PNS abnormalities improve with weight restoration, although recovery trajectories remain variable [28].

## 4. Cardiopulmonary Disease in Anorexia Nervosa

### 4.1. Cardiovascular Complications

Cardiovascular abnormalities represent some of the most frequent medical complications of AN, occurring in up to 87% of patients. Clinical manifestations encompass left ventricular atrophy, mitral valve prolapse, bradycardia, hypotension (often orthostatic), QTc prolongation, arrhythmias, pericarditis, ischemic heart disease, and heart failure (HF). Mortality due to cardiovascular causes is particularly elevated, with epidemiological data indicating a two- to threefold higher risk compared with the general population [29,30]. In a large Taiwanese cohort comprising 2081 patients with AN, Tseng et al. reported a 4.8% cumulative incidence of major adverse cardiovascular events (MACEs) over a five-year follow-up period. The risk of HF, structural cardiac abnormalities, and conduction disturbances was greatest shortly after diagnosis, whereas the incidence of ischemic heart disease rose significantly only by the fifth year. Older age and male sex were identified as independent risk factors for adverse cardiovascular outcomes [31].

Bradycardia, reported in 36–95% of patients, and hypotension, observed in up to 85%, correlate inversely with body mass index (BMI) and often necessitate hospitalization when BMI falls below 15 kg/m^2^ [30,32,33,34,35]. Bradycardia likely reflects an adaptive decrease in basal metabolic rate, whereas hypotension arises from autonomic dysregulation characterized by increased vagal tone. QTc prolongation, observed in up to 40% of patients, becomes clinically significant when exceeding 600 ms due to the risk of ventricular tachycardia, torsades de pointes, and sudden cardiac death [34]. Electrolyte abnormalities, particularly hypokalemia and hypomagnesemia, are common precipitants, although QTc prolongation may also occur independently of electrolyte imbalance, implicating autonomic dysfunction. Certain antipsychotic agents may further exacerbate repolarization disturbances [36,37,38].

Structural myocardial alterations are also well documented. Echocardiography often reveals reduced left ventricular mass index proportional to the degree of undernutrition [34,35]. Histopathological findings include mitochondrial swelling, myxoid and lipofuscin accumulation, and interstitial fibrosis [39]. Thiamine deficiency contributes significantly to myocardial dysfunction, and beriberi cardiomyopathy may coexist [40]. Mitral valve prolapse, affecting up to 25% of patients, likely results from disproportion between ventricular chamber size and leaflet dimensions and typically resolves with weight restoration [30]. Pericardial effusion, observed in up to one-third of patients, is associated with thyroid hormone alterations and hypoalbuminemia and generally regresses following nutritional rehabilitation [30,34].

The refeeding phase is a particularly vulnerable period, as the metabolic transition from catabolism to anabolism can trigger refeeding syndrome, typically manifesting with hypokalemia, hypomagnesemia, and hypophosphatemia within the first 10 days of nutritional reintroduction. These shifts impair myocardial energy metabolism and oxygen delivery, predisposing to arrhythmias and HF even in the presence of preserved ejection fraction [32,34,41,42]. Thiamine deficiency may further exacerbate cardiac dysfunction. Proactive supplementation with electrolytes and thiamine during refeeding is therefore essential to prevent these potentially fatal complications [32].

### 4.2. Pulmonary Complications

Pulmonary complications in AN are less common but clinically relevant. Isolated reports describe spontaneous pneumothorax and pneumomediastinum [43]. Proposed mechanisms include malnutrition-induced bullae or bleb formation predisposing to alveolar rupture, as well as acute intrathoracic pressure changes from forceful vomiting [44]. Aspiration pneumonia may also occur in advanced stages, likely secondary to impaired pharyngeal muscle coordination from severe malnutrition. An increased frequency of emphysema has also been noted, with some authors proposing the entity of “nutritional emphysema,” though its pathogenesis and validity remain debated [30,45].

## 5. Liver Disease in Anorexia Nervosa

Although AN is an uncommon cause of liver disease, abnormalities in liver function are frequently observed among hospitalized AN patients [46]. The predominant mechanism involves starvation-induced autophagy, triggered by endoplasmic reticulum stress. Initially adaptive, this process may become excessive with progressive malnutrition, resulting in hepatocellular injury and cell death [47,48]. Additional contributors include secondary hypoxia due to hypovolemia, particularly in the setting of hypotension or cardiac dysfunction, and, in some cases, iron overload [49,50]. In contrast, insulin resistance (IR) does not appear to play a significant role [51].

Elevated serum aminotransferases are the most common liver abnormality, occurring in more than half of patients. These elevations typically reflect increased hepatocyte membrane permeability from autophagy rather than frank necrosis [52,53]. Although usually mild, aminotransferase levels can rise substantially when BMI falls below 13 kg/m^2^ [53,54]. The biochemical profile often resembles metabolic dysfunction-associated steatotic liver disease (MASLD), with alanine aminotransferase (ALT) exceeding aspartate aminotransferase (AST). Rarely, lactate dehydrogenase (LDH) may increase, usually in the context of hepatic hypoxia, and severe hypoglycemia may occur due to depleted glycogen stores [55]. Notably, serum albumin may remain within the normal range despite profound malnutrition, whereas hematocrit-adjusted albumin has been proposed as a more reliable marker of nutritional status and hepatic recovery [50,56,57].

The international normalized ratio (INR) is typically modestly elevated, though more pronounced increases may be observed in patients with marked transaminase abnormalities. These changes are thought to result primarily from impaired vitamin K absorption rather than reduced hepatic synthetic capacity [58]. Standard anticoagulant prophylaxis for venous thromboembolism (VTE) is generally safe, supported by thromboelastography data indicating preserved coagulation function [59]. Nonetheless, severe coagulopathy and persistent thrombocytopenia, even after refeeding, have been reported, highlighting the importance of regular monitoring of hematological and coagulation parameters. Acute liver failure is rare, while isolated cases of portal hypertension have been described [60,61,62].

**Figure 1:**
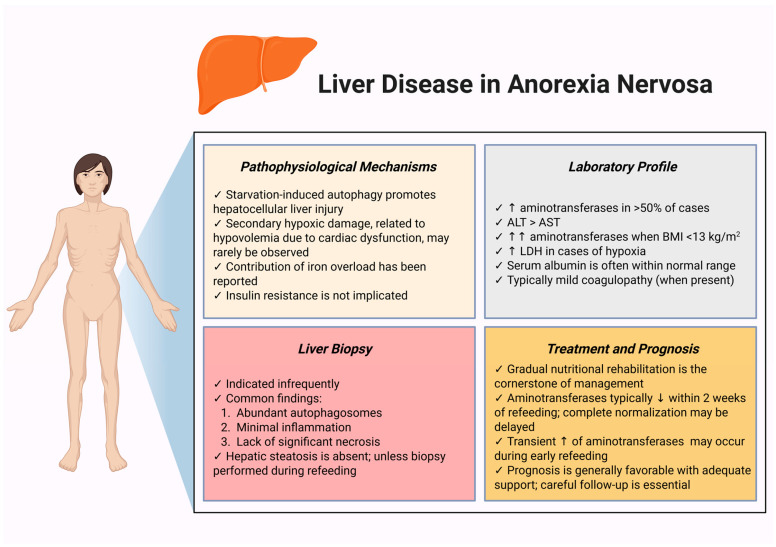
Main characteristics and key approaches to liver disease in anorexia nervosa. Abbreviations: ALT: alanine aminotransferase; AST: aspartate aminotransferase; BMI: body mass index; LDH: lactate dehydrogenase; ↑: increase; ↓: decrease. Created in BioRender. Kounatidis, D. (2025) https://BioRender.com/zs7ulab (assessed on 8 September 2025).

Liver biopsy is seldom required but, when performed, typically demonstrates abundant autophagosomes with minimal inflammation and little or no necrosis. Hepatic steatosis is generally absent in starvation-related hepatitis unless the biopsy is obtained during refeeding, when glucose and fat accumulate in the liver [52]. Gradual nutritional rehabilitation remains the cornerstone of management. Serum aminotransferases usually begin to normalize within two weeks of refeeding, although full recovery may take longer. A mild, transient rise in aminotransferases is often observed during the early refeeding phase [51,52]. Figure 1 presents the key features and clinical management considerations of liver disease in patients with AN.

## 6. Gastrointestinal Disease in Anorexia Nervosa

Gastrointestinal (GI) symptoms are highly prevalent in individuals with AN and may reinforce restrictive eating behaviors, as patients often attribute reduced food intake to abdominal discomfort. Reported manifestations encompass the entire GI tract, including abdominal pain, constipation, and diarrhea, with documented cases of gastroesophageal reflux disease (GERD) and irritable bowel syndrome (IBS) [63,64]. More severe complications, such as gastroparesis and, less commonly, acute gastric dilatation (particularly following binge episodes) have also been observed. Pancreatitis may arise in severely malnourished patients, potentially as a result of hypoperfusion, duodenogastric reflux, or pancreatic atrophy [63,65]. Overly aggressive refeeding may exacerbate these GI complications. Notably, early-life GI disturbances, like persistent constipation or diarrhea, have been linked to subsequent development of AN, though the contribution of shared familial or genetic factors cannot be excluded [66]. While symptoms such as constipation and abdominal pain generally improve with nutritional rehabilitation, complaints of dyspepsia may persist, with psychological distress emerging as a significant predictor of ongoing GI morbidity [67].

## 7. Kidney Disease in Anorexia Nervosa

**Figure 2:**
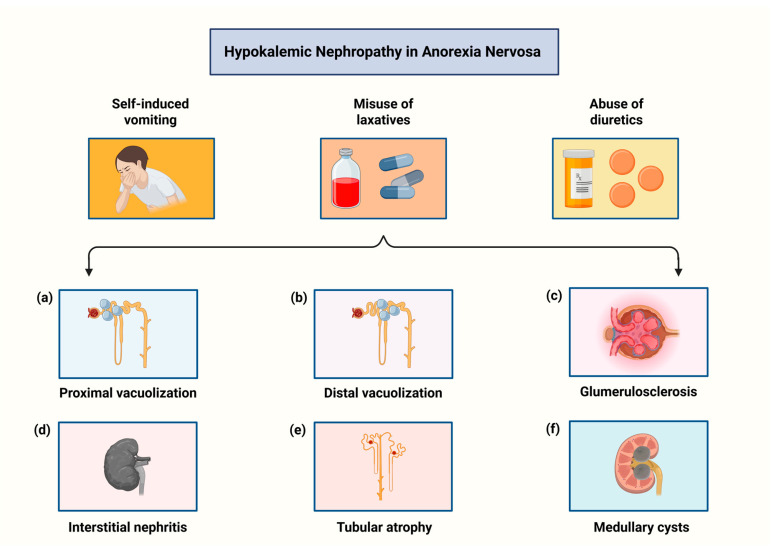
The histopathological spectrum of hypokalemic nephropathy in anorexia nervosa. Created in BioRender. Kounatidis, D. (2025) https://BioRender.com/4tnmh4k (assessed on 1 November 2025).

In a cohort of 395 adolescents with newly diagnosed AN, Gurevich et al. reported impaired kidney function in nearly 37% of patients [68]. Renal complications in AN are primarily driven by electrolyte disturbances and volume depletion, with hypokalemia representing the key pathogenic factor. Hypokalemia commonly arises from self-induced vomiting, laxative misuse, or diuretic abuse and is particularly prevalent in the binge-eating/purging subtype compared with the restricting subtype [68,69,70]. Severe, acute hypokalemia in the context of intravascular depletion may precipitate hypotension, reduced renal perfusion, and acute kidney injury (AKI). During refeeding syndrome, marked hypophosphatemia (serum phosphorus <0.3 mmol/L) can further exacerbate AKI through rhabdomyolysis [69,70,71,72]. Moreover, chronic hypokalemia may induce structural renal injury, termed hypokalemic nephropathy, histologically characterized by vacuolization of proximal tubular epithelial cells, with distal tubular involvement occurring less frequently. Additional reported abnormalities include non-specific glomerulosclerosis, interstitial nephritis, tubular atrophy, and medullary cyst formation. Notably, correction of hypokalemia may reduce cyst burden [69]. Figure 2 depicts the spectrum of histopathological changes associated with hypokalemic nephropathy in AN.

Other electrolyte disturbances further exacerbate renal dysfunction in AN. Hypomagnesemia, frequently coexisting with hypokalemia, often reflects diuretic or laxative misuse compounded by nutritional deficiencies [69,70,71,72,73]. Hyponatremia, resulting from excessive water intake or volume depletion, can trigger inappropriate vasopressin secretion, perpetuating water retention and electrolyte imbalance. Interestingly, this process may be aggravated by concomitant antipsychotic therapy [69]. Beyond electrolyte-mediated injury, nephrocalcinosis, defined by calcium oxalate and/or calcium phosphate deposition within the renal parenchyma, has been reported, with malnutrition and chronic volume depletion implicated in its pathogenesis. Nephrolithiasis may also occur, commonly as ammonium urate calculi promoted by dehydration. Collectively, these abnormalities place patients with AN at risk for both AKI and chronic kidney disease (CKD), with some cases progressing to end-stage renal disease (ESRD) [73,74]. Accurate assessment of renal function in AN remains challenging. Severe muscle wasting reduces creatinine generation, limiting the reliability of creatinine-based estimations of glomerular filtration rate (eGFR). The potential utility of cystatin C, either alone or incorporated into Chronic Kidney Disease Epidemiology Collaboration (CKD-EPI) equations, warrants further investigation as a more accurate biomarker in this population [69].

## 8. Hormonal Abnormalities in Anorexia Nervosa

### 8.1. Hypothalamic–Pituitary–Adrenal (HPA) Axis Dysregulation

Patients with AN, particularly those with the binge–purge subtype, frequently display sustained overactivity of the hypothalamic–pituitary–adrenal (HPA) axis, reflected by elevated levels of corticotropin-releasing hormone (CRH) and adrenocorticotropic hormone (ACTH) [75]. A rare case of ACTH deficiency with secondary adrenal insufficiency has also been documented [76]. Excessive CRH and ACTH secretion contributes to hypercortisolemia, with affected individuals often exhibiting increased salivary and 24 h urinary cortisol levels [77]. The cortisol awakening response is typically exaggerated, especially in the binge–purge subtype compared with the restrictive subtype [78]. In contrast, hair cortisol concentration, a biomarker of long-term cortisol exposure, does not appear elevated and shows weak correlation with urinary cortisol output, possibly indicating altered cortisol incorporation or enzyme dysregulation secondary to severe malnutrition [79]. Weight restoration generally leads to normalization of circulating cortisol levels; however, persistent HPA axis abnormalities have been reported. Schmalbach et al. observed that, despite normalized basal cortisol, individuals with AN demonstrated a blunted cortisol response to acute stress, a pattern more closely associated with residual eating-disorder psychopathology and psychological distress than with BMI [80]. Pharmacological suppression of cortisol is not recommended, given the absence of proven benefit and the potential risks of adrenal insufficiency and further weight loss [81].

### 8.2. Growth Hormone–Insulin-like Growth Factor 1 (GH–IGF-1) Axis Dysregulation

Prolonged starvation in AN is characterized by increased growth hormone (GH) secretion and pulsatility, accompanied by markedly reduced circulating insulin-like growth factor 1 (IGF-1) levels. This paradoxical pattern likely reflects a compensatory adaptation aimed at maintaining euglycemia through enhanced gluconeogenesis and promoting lipolysis. In AN, however, GH hypersecretion occurs in the context of GH resistance, mediated by reduced insulin concentrations and elevated fibroblast growth factor 21 (FGF21), both of which impair GH receptor signaling and hepatic IGF-1 synthesis [77,81]. Growth hormone secretagogue receptor (GHSR) signaling has also been implicated in circadian adaptation during periods of food restriction. In a murine model of AN, GHSR-deficient mice exhibited impaired circadian adjustment, with diminished daytime and pre-prandial activity [82]. Although premorbid stature in AN is generally normal, adult height often remains below midparental expectations. Partial catch-up growth may occur with nutritional rehabilitation; however, final stature depends on chronological age, bone age, growth velocity during hospitalization, and concurrent changes in luteinizing hormone (LH) secretion [83]. Growth hormone therapy has demonstrated potential benefits in patients with persistent AN-related growth failure, increasing linear growth velocity compared with placebo over a 12-month period [84].

### 8.3. Hypothalamic–Pituitary–Gonadal (HPG) Axis Dysregulation

Menstrual irregularities are highly prevalent in AN, with functional hypogonadotropic hypogonadism (FHH) most commonly manifesting as amenorrhea, reported in up to 85% of cases. Loss of adipose tissue leads to leptin deficiency, disrupting kisspeptin-mediated stimulation of hypothalamic gonadotropin-releasing hormone (GnRH) neurons. This impairs pulsatile GnRH secretion and subsequently reduces LH and follicle-stimulating hormone (FSH) output. As a result, ovarian estradiol and testicular testosterone production decline [77,81]. Weight restoration typically reverses FHH, though menstrual recovery may take up to one year, and approximately 20% of women fail to resume menses despite normalization of body weight [81,85]. Among weight-restored individuals, higher estradiol concentrations and greater LH/FSH responsiveness to GnRH predict menstruation resumption. Specific endocrine thresholds, such as the presence of at least two LH pulses within four hours and an LH/GnRH response ≥33 IU/L, have demonstrated strong predictive value independent of body weight [86]. Despite amenorrhea, conception remains possible; thus, early fertility counseling is recommended due to increased obstetric risks, including low birth weight, preterm delivery, and intrauterine growth restriction [87,88].

### 8.4. Hypothalamic-Pituitary-Thyroid (HPT) Axis Dysregulation

Typical clinical manifestations of AN, including dry skin, hypothermia, hypotension, and bradycardia, frequently reflect underlying thyroid dysfunction. Severe malnutrition often induces a non-thyroidal illness syndrome (NTIS), characterized by reduced triiodothyronine (T3), elevated reverse T3 (rT3), and thyroid-stimulating hormone (TSH) and free thyroxine (fT4) levels within the low-normal range [89,90]. Suppression of hypothalamic-pituitary-thyroid (HPT) axis activity, indicated by reduced fT3 and a decreased fT3/fT4 ratio, appears more pronounced in individuals with severe depressive symptoms [91]. An fT3/fT4 ratio below 2.0 has been proposed as a biomarker distinguishing NTIS from central hypothyroidism in adolescents [92]. Thyroid hormone alterations generally improve with nutritional rehabilitation, although complete normalization is not universal. Animal studies suggest that early nutritional support may prevent T3 decline during fasting by modulating deiodinase activity and leptin signaling [93]. Exogenous thyroid hormone replacement is not recommended, as it may disrupt adaptive hypometabolism and heighten cardiovascular risk [81].

### 8.5. Posterior Pituitary Hormone Dysregulation

Individuals with AN may exhibit impaired osmoregulation, as evidenced by lower plasma sodium and osmolality, elevated antidiuretic hormone (ADH) concentrations, and diminished urine-concentrating capacity. ADH responses appear dysregulated, with persistently dilute urine particularly in chronic illness [94]. In contrast, copeptin, used as a stable surrogate marker of ADH, does not differ between medically stable patients with AN and healthy controls, suggesting that ADH activity may not be elevated under normohydrated conditions [95]. Notably, central diabetes insipidus can emerge during the refeeding phase, with MRI findings of absent posterior pituitary high signal (PPHS) indicating ADH granule depletion [96,97].

Oxytocin dysregulation has also been implicated in AN pathophysiology, with reduced serum and cerebrospinal fluid levels reported [81,98]. Elevated postprandial oxytocin concentrations have been associated with heightened anxiety and depressive symptoms, independent of cortisol and largely unaffected by leptin status [99]. Conversely, other studies have described postprandial oxytocin declines, more pronounced in atypical AN, potentially contributing to appetite dysregulation [100]. Associations between oxytocin and psychopathology may vary by subtype, with restrictive AN showing broadly negative correlations across symptom domains, while binge–purge AN is more closely linked to depressive symptoms [101]. These findings have prompted investigation of intranasal oxytocin (IN-OT) as a therapeutic strategy. A pilot inpatient trial reported reductions in eating-related preoccupations, cognitive rigidity, and salivary cortisol responses. However, a subsequent multicenter randomized controlled trial (RCT) failed to replicate these results, suggesting that treatment effects may depend on baseline symptom severity and comorbid psychiatric profiles [102,103].

### 8.6. Adipokine Dysregulation

Fat mass depletion in AN may produce significant alterations in adipokine secretion. Leptin, a key regulator of satiety, neuroendocrine function, and metabolic homeostasis, is reduced, initiating starvation-adaptive processes that operate through both threshold-dependent “switches” and graded, tissue-specific modulation [104]. Low leptin contributes to hyperactivity, a relationship absent post-recovery, when activity aligns more closely with core psychopathology [105,106]. Ghrelin signaling is impaired via increased methylation of the growth hormone secretagogue receptor 1a (GHSR1a) promoter, consistent with “ghrelin resistance,” explaining the lack of compensatory hyperphagia despite elevated circulating and exogenous ghrelin, while anxiety, food preoccupation, and altered reward processing persist [107]. Elevated liver-expressed antimicrobial peptide 2 (LEAP2), an endogenous GHSR antagonist, may reinforce this selective resistance, highlighting complex interactions between metabolic signals and neural reward circuits [108]. Circulating adiponectin, which is typically elevated during caloric restriction, is influenced by metabolic and inflammatory mediators such as insulin, resistin, triglycerides, and interleukin (IL)-6. Its concentrations correlate positively with GH pulsatility but remain unaltered by short-term GH elevations, suggesting regulation primarily by fat mass rather than by acute endocrine fluctuations [109,110].

## 9. Musculoskeletal Disease in Anorexia Nervosa

Malnutrition in subjects with AN is closely correlated with generalized fatigue and attenuated physical endurance, mainly resulting from skeletal muscle atrophy. Nutritional deficits, electrolyte imbalances, metabolic alterations, and hypercortisolemia contribute to the selective degeneration of type II muscle fibers [111,112]. This catabolic milieu can lead to functional impairments, including difficulties in maintaining posture, and a heightened risk of falls. Skeletal involvement in AN is predominantly characterized by a reduction in bone mineral density (BMD), which can progress from osteopenia to osteoporosis, thereby predisposing to fragility fractures. Dual-energy X-ray absorptiometry (DEXA) studies have demonstrated markedly reduced BMD in individuals with AN, often within the osteoporotic range [113,114]. The pathophysiology underlying these bone abnormalities is multifactorial. FHH favors estrogen deficiency, while changes in GH secretion and reduced IGF-1 impair osteoblastic bone formation. Additional contributing factors include vitamin D deficiency, depletion of calcium and phosphorus, persistent hypercortisolemia, and dysregulation of adipokines such as leptin and adiponectin. In addition, serum osteocalcin concentrations are decreased, reflecting reduced osteoblastic activity and a consequent imbalance between bone formation and resorption [115,116]. Histological and imaging studies further indicate a pathological shift in bone marrow composition from osteogenesis toward adipogenesis, thereby accelerating bone loss [117].

## 10. Cutaneous Manifestations in Anorexia Nervosa

**Figure 3:**
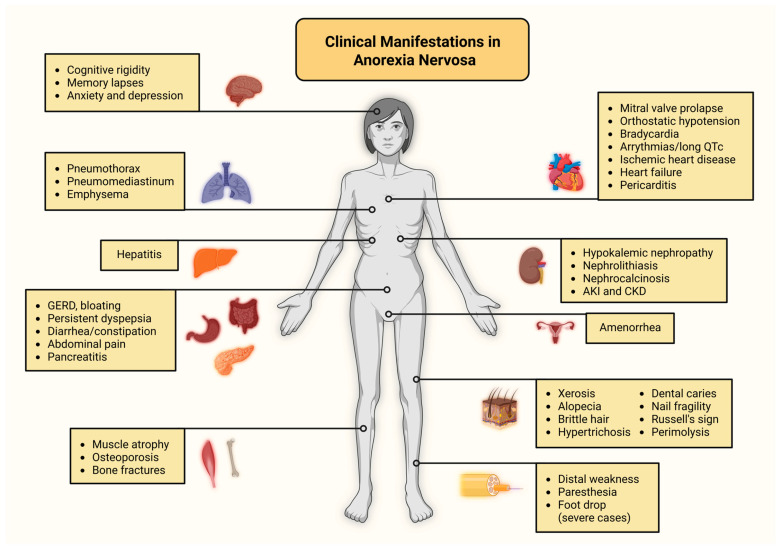
Clinical consequences of anorexia nervosa. Abbreviations: AKI: acute kidney injury; CKD: chronic kidney disease; GERD: gastroesophageal reflux disease; LV: left ventricular. Created in BioRender. Kounatidis, D. (2025) https://BioRender.com/3hfpuek (assessed on 6 September 2025).

Skin signs are common in advanced AN, particularly when BMI falls below 16 kg/m^2^ [118]. A functional classification identifies four principal categories: (i) cutaneous signs of starvation, (ii) lesions related to self-induced vomiting, (iii) injuries from self-inflicted behaviors, and (iv) changes associated with substance misuse, including laxatives and diuretics. Often, multiple mechanisms contribute to individual clinical findings [119]. Xerosis, or dry skin, is among the most prevalent features, attributed to reduced sebaceous gland activity and altered lipid composition. Dehydration from purging behaviors, diuretic or laxative use, and excessive hygiene further exacerbates this condition [119,120]. Hair abnormalities are also frequent and include alopecia, brittle or thinning hair, and lanugo-like growth, with the latter potentially related to decreased 5-α-reductase activity or concurrent hypothyroidism. Additional cutaneous changes include eczema, acne, nail fragility, and pigmentary alterations such as hyperpigmentation, carotenoderma, and acrocyanosis [119,121,122]. Carotenoderma is typically linked to a high intake of carotenoid-rich vegetables, while acrocyanosis reflects underlying vascular dysregulation. Pruritus is frequently reported and may be generalized, influenced by hormonal imbalance, and impaired thermoregulation. Characteristic signs include Russell’s sign, manifested as callused knuckles from repeated self-induced vomiting, and perimolysis, characterized by enamel erosion resulting from chronic exposure to gastric acid. Among the most common dermatological findings in AN are xerosis, alopecia, dental caries, brittle hair, and nail fragility, while features such as hypertrichosis, Russell’s sign, perimolysis, and self-inflicted lesions are considered more specific to the disorder. Early recognition of these manifestations may facilitate identification of patients who do not openly disclose disordered eating behaviors [123,124]. Figure 3 summarizes the common clinical features observed in AN.

## 11. Hematological Alterations in Anorexia Nervosa

Hematological assessment represents an essential component of the clinical evaluation of AN, as complete blood count (CBC) abnormalities commonly reflect the systemic effects of malnutrition. The most frequently observed alterations include anemia, leukopenia, and thrombocytopenia, all of which generally improve with nutritional rehabilitation [125]. The prevalence and severity of these changes correlate closely with the degree and duration of protein–energy malnutrition, as reflected by BMI and phase angle measurements [126]. Reported frequencies vary across studies, likely owing to differences in sample characteristics and nutritional status. Earlier investigations from Europe and the United States identified anemia as the most frequent abnormality, followed by leukopenia and, less often, thrombocytopenia [127,128]. More recent data, however, indicate that leukopenia predominates, while a global meta-analysis estimated the overall prevalence of anemia in AN at 44.8% [129,130]. Bone marrow biopsies typically reveal hypocellularity with adipose replacement and, in some cases, gelatinous marrow transformation (GMT) [131]. Despite these findings, bone marrow biopsy is not recommended in routine clinical practice [129].

During refeeding, hematological indices may transiently deteriorate, often reaching their nadir between days five and ten of hospitalization. This decline tends to be more pronounced in restrictive-type AN or in the presence of intercurrent infection and may necessitate transfusion support [132]. Anemia is usually normocytic and normochromic, though cases of macrocytosis have been reported [127,129]. The relationship between neutropenia and infection risk in AN remains uncertain. Individuals admitted with a BMI below 12 kg/m^2^ appear particularly vulnerable, and earlier studies suggested that impaired bactericidal activity and reduced polymorphonuclear cell adherence may compound this risk [133,134]. Nevertheless, evidence for a higher incidence of infections in AN is inconclusive. Clinical vigilance is therefore critical, as infections may present atypically, with absent fever or blunted inflammatory responses [135].

**Figure 4:**
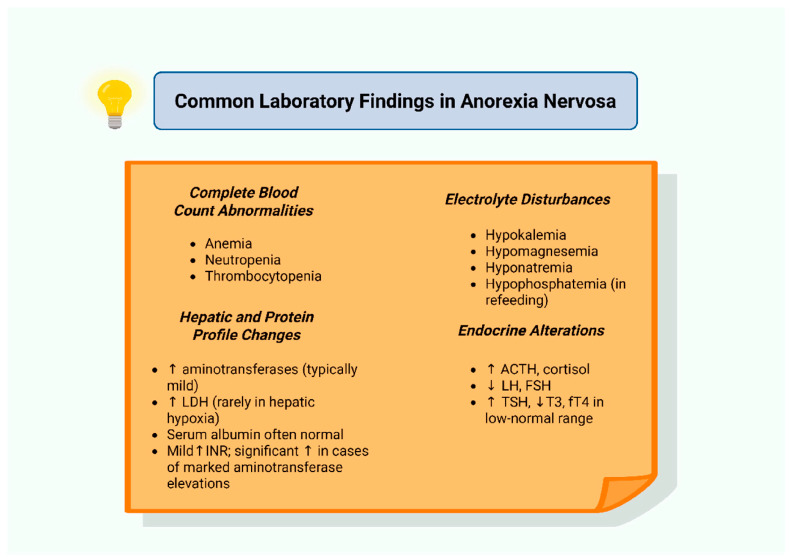
Common laboratory findings in anorexia nervosa. Abbreviations: ACTH: adrenocorticotropic hormone; fT4: free thyroxine; FSH: follicle-stimulating hormone; INR: international normalized ratio; LDH: lactate dehydrogenase; LH: luteinizing hormone; T3: triiodothyronine; TSH: thyroid-stimulating hormone; ↑: increase; ↓: decrease. Created in BioRender. Kounatidis, D. (2025) https://BioRender.com/ye572om (assessed on 7 September 2025).

In cases of severe neutropenia, the therapeutic role of granulocyte colony-stimulating factor (G-CSF) remains poorly defined but may be considered in protracted or complicated presentations [62,130,136]. Thrombocytopenia can also occur in a subset of patients and may be accompanied by increased platelet distribution width (PDW), a parameter potentially reflecting both illness duration and the rate of weight loss [137]. In instances of severe hepatic dysfunction, impaired thrombopoietin synthesis may further contribute to reduced platelet counts [138]. Figure 4 summarizes the principal hematological and laboratory abnormalities observed in AN.

## 12. Emerging Pathophysiological Pathways in Anorexia Nervosa: The Role of Immunity and the Gut–Brain Axis

Beyond the well-characterized systemic adaptations associated with AN, accumulating evidence implicates dysregulation of immune function and disturbances within the gut–brain axis as significant contributors to its complex pathophysiology. These emerging frameworks highlight the intricate interplay between peripheral metabolic and inflammatory processes and central neural regulation of appetite, mood, and cognition. A deeper understanding of these interactions may clarify how AN influences and is maintained by both immune and neurobiological mechanisms and could guide the development of more targeted prevention and treatment strategies. The following subsections synthesize current data on the role of immune alterations and gut–brain communication in the onset, development, and progression of AN.

### 12.1. Immunological Alterations in Anorexia Nervosa

Nutritional deprivation has long been implicated in immune dysregulation, and accumulating data suggest that AN is accompanied by distinct alterations in both innate and adaptive immunity [139,140]. However, the available evidence remains heterogeneous and is often limited by small cohort sizes. Components of innate immunity appear blunted, although the contribution of specific subsets such as macrophages and dendritic cells has yet to be fully characterized [141]. Circulating levels of macrophage inhibitory cytokine-1 (MIC-1) tend to rise during starvation and decline following refeeding, a fluctuation that may be potentiated by hyperinsulinemia [142,143]. Natural killer (NK) cell counts are generally reduced, while their cytotoxic function appears largely preserved [141,144].

Adaptive immune responses are likewise perturbed. Although the proliferative capacity of T cells is usually maintained, an elevated CD4^+^/CD8^+^ ratio is frequently observed, largely due to selective depletion of CD8^+^ subsets, a shift that could impair antiviral defenses [145]. Overexpression of the C-X-C chemokine receptor type 4 (CXCR4) on CD4^+^ T cells has been associated with both psychological distress and nutritional status in adolescents with AN [146], whereas regulatory T cell (Treg) function appears relatively preserved [147]. Findings regarding humoral immunity are ambiguous. B cell counts may be normal or elevated, with restrictive-type AN showing expansion of antigen-experienced subsets alongside depletion of immunoregulatory populations [148]. Antibody titers are typically reduced or within normal limits, yet vaccine responsiveness remains intact [141,149]. Reports also suggest aberrant T–B cell crosstalk, and a decreased neutrophil-to-lymphocyte ratio (NLR) has been proposed as a potential marker of disease severity [141,150].

Cytokine profiling has yielded similarly variable results, although most studies report elevated concentrations of pro-inflammatory cytokines, including IL-1β, IL-6, and IL-8 [151]. Prostaglandin E_2_ (PGE_2_) has been implicated in IL-1β-driven anorexic behavior, albeit with strain-specific variability [152]. Findings regarding tumor necrosis factor-alpha (TNF-α) remain inconsistent. For instance, one study demonstrated in vitro release comparable to controls, whereas lipopolysaccharide (LPS)-stimulated secretion appeared attenuated, possibly reflecting reduced monocyte availability. Other investigations reported increased IL-6 and transforming growth factor-beta (TGF-β) without significant TNF-α alterations, while some data support spontaneous TNF-α production [151,153]. Reduced interferon-gamma (IFN-γ) secretion by lymphocytes has also been described, though findings are not uniform across studies [151,154].

Importantly, immune abnormalities may emerge even in the early stages of AN, with improvements in cytokine profiles during treatment paralleling reductions in depressive symptoms [155]. Restrictive-type AN may not initially exhibit overt pro-inflammatory activation; nonetheless, disturbances in immune-related mediators such as C-X-C motif chemokine ligand 1 (CXCL1) and matrix metalloproteinase-8 (MMP-8) have been documented [156]. Complement activity remains underexplored, although reduced C3 levels have been correlated with disease severity and improve with weight restoration [157]. Oxidative stress also appears to modulate immune function, as indicated by reduced serum uric acid and total antioxidant capacity, both of which normalize following nutritional rehabilitation [158,159].

While some immune abnormalities in AN resemble those observed in primary malnutrition, the overlap is only partial, suggesting that caloric restriction alone does not fully account for the immunological phenotype of AN [141]. Endocrine factors, particularly adipokines such as leptin and adiponectin, likely exert additional modulatory effects on immune responses [160]. Collectively, the available evidence supports an immunological contribution to the pathophysiology of AN, although its precise clinical significance remains incompletely defined. The partially autoimmune potential of AN is further suggested by its co-occurrence with immune-mediated diseases, particularly those affecting the gastrointestinal tract, such as celiac disease (CeD) and inflammatory bowel disease (IBD). In the case of CeD, comparable patterns of immune dysregulation have been described, albeit mediated through distinct mechanisms of aberrant immune activation and inflammatory signaling [161]. On the other hand, the relationship between AN and IBD remains less consistent. Population-based data and genetic analyses yield divergent findings regarding both the strength and direction of association between AN and specific IBD subtypes, namely Crohn’s disease (CD) and ulcerative colitis (UC). Overall, the mechanistic basis underlying the interplay between AN and immune-mediated GI disorders remains unclear and warrants further investigation [162,163,164]. Additional immune-related phenomena include hepatic cytolysis linked to anti-hypoxia-inducible factor 1-alpha (HIF-1α) autoantibodies, and reactivation of latent human herpesviruses 6 and 7 (HHV-6, HHV-7) leading to pityriasis rosea. Interestingly, reduced salivary immunoglobulin M (IgM) concentrations have been correlated with increased dental plaque accumulation in subjects with AN [50,165,166,167].

### 12.2. The Gut–Brain Axis in Anorexia Nervosa

The gut microbiota, comprising bacteria, viruses, fungi, and archaea, and its genomic counterpart, the gut microbiome, play a central role in maintaining host homeostasis. Disruption of this balance gives rise to gut dysbiosis, which in AN is marked by increased intestinal permeability, reduced microbial diversity, and distinct compositional alterations. These include elevated levels of *Enterobacterales*, *Proteobacteria*, and *Methanobrevibacter smithii*, alongside reductions in *Faecalibacterium prausnitzii* and *Firmicutes* [168,169,170]. Beyond bacterial shifts, patients with AN demonstrate diminished viral–bacterial interactions and enrichment of microbial pathways linked to neurotransmitter degradation, suggesting that dysbiosis may contribute to neurobiological processes underlying the disorder [171]. Within this context, the gut–brain axis (GBA), a bidirectional communication network integrating immune, hormonal, and metabolic signals, has become a major focus of contemporary research [172,173].

Loss of intestinal barrier integrity facilitates the translocation of LPS from Gram-negative bacteria into the circulation via disrupted tight junction proteins, a phenomenon commonly referred to as “leaky gut”. This process elicits an immune response marked by the release of pro-inflammatory cytokines, including IL-6 and TNF-α, which can cross the blood–brain barrier and promote neuroinflammation [170]. The hypothalamus, enriched in cytokine receptors and central to appetite regulation, is particularly sensitive to these signals. Toll-like receptor 2 (TLR-2) and toll-like receptor 4 (TLR-4) activation of microglia, leading to NLR family pyrin domain-containing protein 3 (NLRP3) inflammasome activation, provides a key pathway through which peripheral immune disturbances may disrupt hypothalamic function [174,175,176]. The role of the hypothalamus in weakening immune defense and further destabilizing intestinal barrier integrity is underscored by the chronic hyperactivity of the HPA axis observed in AN, manifested in elevated cortisol secretion, particularly in the binge–purge subtype [177].

Metabolic signaling through short-chain fatty acids (SCFAs) constitutes another mechanism shaping neuroinflammation. SCFAs, such as butyrate, are generated by bacterial fermentation of dietary fibers, and patients with AN have been shown to display reduced fecal concentrations of butyrate and acetate compared with healthy controls [178]. Diminished butyrate production has also been associated with lower levels of brain-derived neurotrophic factor (BDNF), impairing neuronal growth, survival, and plasticity, and thereby contributing to disturbances of appetite regulation and mood [179]. Other microbiota-derived metabolites, including caseinolytic peptidase B (ClpB), can mimic the anorexigenic hormone α-melanocyte-stimulating hormone (α-MSH), further enhancing appetite suppression [172]. In addition, severely ill patients may demonstrate reduced circulating concentrations of diazepam-binding inhibitor (DBI). A decline in DBI weakens inhibitory control over anorexigenic signals such as growth differentiation factor 15 (GDF15) and lipocalin-2 (LCN2), thereby reinforcing restrictive eating behaviors [180].

Altered tryptophan metabolism represents an additional pathway through which the gut influences brain function in AN. Gut microbes divert tryptophan away from serotonin (5-HT) synthesis, reducing the substrate available for neurotransmitter production. Combined with dietary restriction, this leads to further 5-HT depletion, increasing vulnerability to mood disturbances and cognitive deficits [181]. Autonomic dysregulation may boost these effects, as the gut–vagus nerve pathway serves as a critical conduit for microbial signaling to the brain. Evidence from animal studies indicates that vagal input can modulate dopamine release, inflammatory responses, and emotional behavior, although human data remain mixed [182].

**Figure 5:**
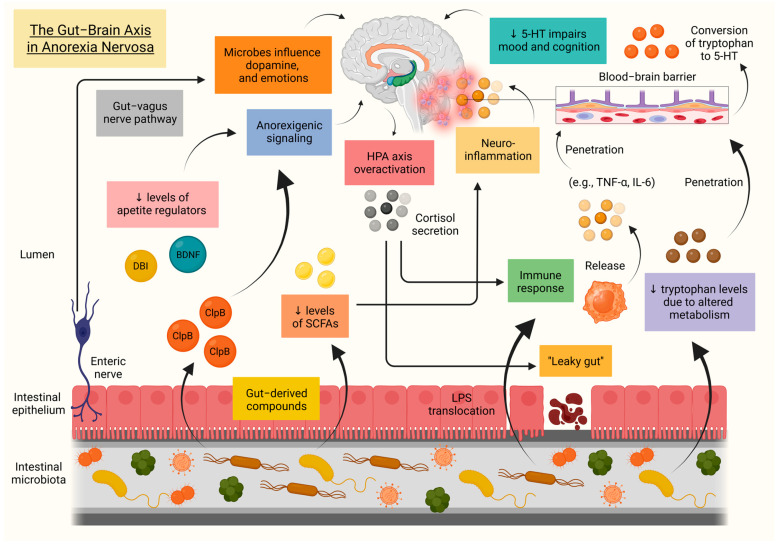
The “busy” gut–brain axis in anorexia nervosa. This figure depicts the role of the GBA in the development and progression of AN, through interconnected neuroimmune, neuroendocrine, and neurotrophic pathways. A central event in this cascade is the loss of intestinal barrier integrity, commonly referred to as “leaky gut”, which permits the translocation of LPS into the systemic circulation, leading to peripheral immune activation and promoting neuroinflammation. These effects are exacerbated by reduced production of SCFAs and by sustained hypercortisolemia resulting from chronic hyperactivation of the HPA axis. The bacterial chaperone protein ClpB exerts anorexigenic effects via molecular mimicry with α-MSH, which are compounded by decreased circulating levels of appetite regulators, such as DBI and BDNF. Lastly, disturbances in tryptophan metabolism reduce serotonin bioavailability, while impaired autonomic regulation, particularly through altered vagus nerve signaling, further contributes to disease progression. Abbreviations: 5-HT: 5-hydroxytryptamine; α-MSH: α-melanocyte-stimulating hormone; AN: anorexia nervosa; BDNF: brain-derived neurotrophic factor; ClpB: caseinolytic peptidase B; DBI: diazepam-binding inhibitor; GBA: gut–brain axis; HPA: hypothalamic–pituitary–adrenal; IL-6: interleukin-6; LPS: lipopolysaccharides; SCFA: short-chain fatty acid; TNF-α: tumor necrosis factor-alpha; ↓: decrease. Created in BioRender. Kounatidis, D. (2025) https://BioRender.com/o4pd1rv (assessed on 7 September 2025).

Overall, current evidence indicates that gut dysbiosis in AN disrupts neuroimmune, neuroendocrine, and neurotrophic processes through the GBA. This interplay positions the gut microbiome not only as a potential biomarker of disease progression but also as a promising therapeutic target. Figure 5 presents the role of the GBA in the development and progression of AN.

## 13. Anorexia Nervosa Management: Current Strategies and Therapeutic Perspectives

### 13.1. Current Strategies

The management of AN requires a coordinated, multidisciplinary approach integrating the expertise of psychiatrists, psychologists, and medical–nutrition specialists. Nutritional education should be initiated at the outset and reinforced throughout treatment, with the goal of establishing sustainable, health-promoting eating behaviors. The involvement of registered dietitians is essential to ensure safe and progressive weight restoration. Traditional refeeding protocols typically began with approximately 1200 kcal/day; however, more recent strategies employing 1400–1600 kcal/day have been shown to be safe when patients are closely monitored. Vigilance for refeeding syndrome remains paramount, given its potential lethality during early nutritional rehabilitation. Electrolyte levels must be assessed regularly, and supplementation with thiamine, riboflavin, vitamin D, calcium, and other essential micronutrients should be administered. A realistic weight-gain target is approximately 0.5 kg per week [183]. Table 1 depicts recent studies of nutritional management in patients with AN.

Psychotherapy remains the cornerstone of AN treatment. Evidence-based modalities include family-based interventions, cognitive behavioral therapy (CBT), and the Maudsley model for adults. Emerging approaches, such as the provision of calorie-dense foods in prepackaged formats, have been investigated to enhance dietary adherence. Acceptance and commitment therapy (ACT), which emphasizes restructuring maladaptive cognitions, fostering intrinsic motivation, and reinforcing value-driven behaviors, has also garnered increasing attention. On the contrary, pharmacological interventions have demonstrated limited efficacy. Antidepressants, in particular, yield minimal clinical benefit. To date, no effective pharmacological treatment for AN has been established, a therapeutic gap that has prompted intensified research into alternative strategies [194,195].

### 13.2. Challenges and Therapeutic Perspectives

#### 13.2.1. Atypical Antipsychotics

The limited efficacy of antidepressants in AN has prompted growing interest in the therapeutic potential of atypical antipsychotics. Among second-generation agents, olanzapine and aripiprazole appear particularly promising in cases of severe or treatment-resistant illness. Although the evidence remains inconsistent, recent studies identify olanzapine as a potential candidate, with reported benefits including weight restoration and attenuation of obsessive food-related thoughts. Qualitative research has described olanzapine treatment as a liminal “rite of passage”, offering adolescents with AN a sense of autonomy and empowerment, while simultaneously underscoring tensions around weight gain and communication with parents and clinicians [196].

#### 13.2.2. Psychedelics, Dronabinol and Ketamine

Psychedelic compounds have recently gained attention as potential therapeutic strategies for AN. Psilocybin, a serotonergic agent derived from fungi, may modulate mood, perception, and cognition and has been proposed as a candidate treatment. In a phase 1 open-label feasibility study, women with AN received a single 25 mg dose of psilocybin accompanied by psychological support. The intervention was well tolerated, with only two brief, asymptomatic episodes of hypoglycemia, and participants generally considered the treatment acceptable [197]. Supporting these findings, a large observational study of 6612 individuals with EDs reported self-perceived symptom improvement specifically associated with cannabis or psychedelic use, although such anecdotal evidence requires confirmation in controlled trials [198]. Evidence is also available for the synthetic cannabinoid agonist dronabinol, derived from a randomized, double-blind, placebo-controlled crossover study involving twenty-five women aged over 18 years with AN of at least five years’ duration. Over the four-week treatment period, dronabinol administration resulted in small but statistically significant weight gain (*p* < 0.01) and was generally well tolerated [199].

Research has likewise examined ketamine, an N-methyl-D-aspartate (NMDA) receptor antagonist with established antidepressant efficacy. Beyond its mood-regulating properties, ketamine may enhance neuroplasticity, neurogenesis, and hippocampal integrity while mitigating neuroinflammation and depressive symptoms that impede recovery in AN. When combined with psychotherapy, ketamine could further promote cognitive flexibility and therapeutic engagement. Early findings, derived primarily from case studies, are encouraging. Nevertheless, further research is required to establish optimal dosing regimens, clinical efficacy, and long-term safety, particularly given the ongoing concerns surrounding ketamine use and potential safety risks [200].

#### 13.2.3. Anti-Inflammatory and Mitochondria-Targeted Strategies

Anti-inflammatory interventions, particularly those involving cyclooxygenase (COX) inhibition, have been investigated for their potential to mitigate anxiety and mood disturbances while promoting neural resilience and synaptic integrity [195]. Another attractive therapeutic target is growth differentiation factor 15 (GDF15), a stress-responsive cytokine implicated in mitochondrial oxidative stress and energy homeostasis. Emerging evidence indicates that subsets of patients with AN display elevated circulating GDF15 levels [201]. In conditions such as cancer cachexia and chemotherapy-induced anorexia, increased GDF15 concentrations have been associated with appetite suppression through the activation of emetic and nausea-related pathways [201,202]. These findings have led to the hypothesis that GDF15 neutralization might ameliorate weight loss in AN. However, experimental studies using genetic ablation or antibody-mediated neutralization of GDF15 in sepsis models have demonstrated negligible effects on appetite or body weight, complicating the translational relevance of this approach [203]. Consequently, it remains uncertain whether GDF15-targeted interventions could yield tangible benefits in promoting weight restoration. The paucity of large-scale, controlled studies highlights the need for further investigation into GDF15 modulation, as delineating its precise role in AN may offer new perspectives for metabolic and behavioral therapeutic strategies.

#### 13.2.4. Recombinant Human Leptin Analogs

As previously discussed, hypoleptinemia in AN may exacerbate weight-related anxieties and obsessive food-related cognitions. Recombinant leptin analogs, particularly metreleptin, have therefore been proposed as potential therapeutic agents targeting both metabolic and affective dysregulation [204]. Originally approved for the management of metabolic complications in lipodystrophy, metreleptin has demonstrated diverse endocrine-regulatory properties, including the restoration of reproductive function in women with hypothalamic amenorrhea and the attenuation of weight-gain-related fear and anxiety [205,206]. A notable case report described a 16-year-old girl with atypical AN who experienced rapid improvement in mood, diminished weight-related phobia, and facilitated hospital discharge following an 11-day course of metreleptin administration [207]. Beyond AN, leptin signaling has also become a focus of investigation in oncology, where leptin antagonists have shown encouraging yet unconfirmed results in clinical settings [208]. Similarly, leptin-based interventions for AN remain largely experimental, as no RCTs have yet been conducted. Moreover, the modest weight reduction observed in some metreleptin-treated cases warrants cautious interpretation and vigilance regarding its catabolic potential. Notably, although metreleptin has been reported to transiently enhance hunger shortly after subcutaneous administration, this effect appears to wane over time and may even be followed by mild weight loss [209]. Taken together, while isolated case reports suggest that metreleptin may offer psychological and metabolic benefits in selected individuals with AN, its clinical application, even in short courses, remains contentious and requires rigorous validation through well-designed clinical trials.

#### 13.2.5. Gut-Directed Therapeutic Interventions

The gut microbiome has emerged as an attractive therapeutic target in AN. Probiotics and prebiotics, acting synergistically, support microbial balance and promote immune, metabolic, and gastrointestinal health. They have demonstrated benefits in type 2 diabetes (T2D), obesity, and IBD [210]. Preliminary findings in AN suggest possible roles in modulating immune activity and alleviating comorbid depression and anxiety [211]. In a randomized, double-blind, placebo-controlled trial, *Lactobacillus reuteri* had no effect on constipation at three months but was associated with greater stool frequency and faster normalization of body weight at six months. Additional benefits included improved BMD and vitamin D levels, suggesting that probiotics may represent a safe adjunct to nutritional rehabilitation in pediatric AN [212]. However, results across studies remain heterogeneous, and larger trials are needed [195].

Fecal microbiota transplantation (FMT) has also been investigated. Transplantation of microbiota from AN patients into mice reduced food intake and locomotor activity while altering microbial profiles and appetite-regulating hormones. Conversely, FMT from healthy donors reversed these effects, while data from meta-analyses support its role in enhancing microbial diversity [213]. Findings to date remain mixed. For example, Kooij et al. reported that FMT from AN patients did not alter cognitive flexibility, anxiety, or dopaminergic signaling in animal models, highlighting the need for more refined experimental approaches [214]. Meanwhile, an ongoing RCT is investigating whether direct colonic administration of SCFAs over six weeks can influence stress reactivity, eating behaviors, BMI, and restrictive patterns in AN [215].

#### 13.2.6. Neuromodulation with Intermittent Theta-Burst Stimulation (iTBS)

Intermittent theta-burst stimulation (iTBS) is a patterned form of non-invasive brain stimulation that delivers bursts of magnetic pulses to modulate cortical excitability and synaptic plasticity. While iTBS has demonstrated efficacy in major depressive disorder and other neuropsychiatric conditions, its use in AN remains exploratory and largely confined to pilot investigations [195]. A current feasibility study is enrolling 66 adolescents and young adults with AN who have shown suboptimal response to conventional care. Participants are randomized to receive either active or sham iTBS in conjunction with standard treatment. The primary outcome measure is four-month treatment retention, with secondary endpoints including changes in symptom severity, quality of life (QoL), and neurobiological indices. The results of this trial are anticipated to inform the methodological and clinical design of future large-scale studies evaluating iTBS as an adjunctive therapeutic strategy in AN [216].

**Figure 6:**
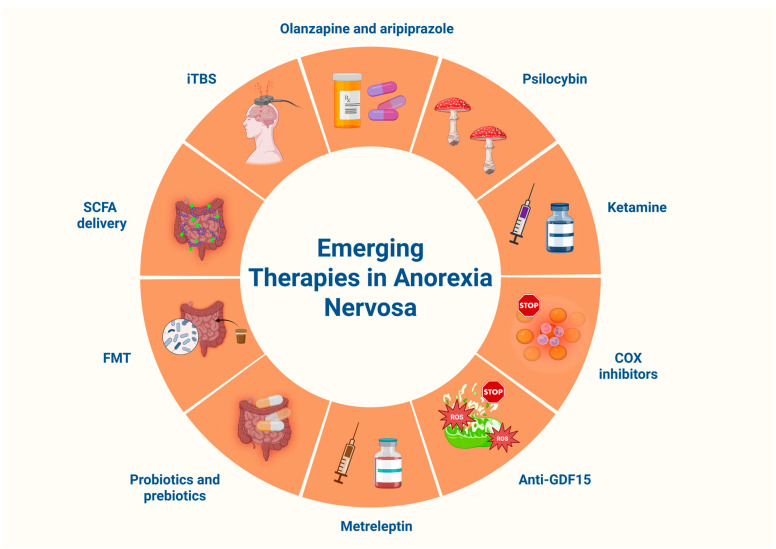
Emerging therapies in anorexia nervosa. Abbreviations: COX: cyclooxygenase; FMT: fecal microbiota transplantation; GDF15: growth and differentiation factor 15; iTBS: intermittent theta-burst stimulation; ROS: reactive oxygen species; SCFA: short-chain fatty acid. Created in BioRender. Kounatidis, D. (2025) https://BioRender.com/jqw1j8l (assessed on 3 November 2025).

Collectively, the emerging interventions discussed above represent innovative efforts to directly modulate the neurobiological underpinnings of AN. Although these strategies hold theoretical and preliminary clinical promise for improving outcomes and enhancing QoL, most remain at preclinical or early translational stages and are not yet suitable for routine clinical implementation. The existing body of research is limited by small sample sizes, lack of randomization, and reliance on uncontrolled or case-based data, thereby precluding firm causal inferences. Of note, the 2023 update of the World Federation of Societies of Biological Psychiatry (WFSBP) guidelines on the pharmacological management of EDs emphasized that olanzapine has not received marketing authorization from any medicinal regulatory agency for the treatment of AN, underscoring the continuing absence of an approved pharmacotherapy for this condition [217]. Figure 6 summarizes the range of emerging therapeutic strategies currently under investigation for AN management.

## 14. Conclusions

AN remains one of the most challenging disorders at the interface of psychiatry, internal medicine, and neuroscience. Its complex pathophysiology, spanning genetic, immunological, and gut–brain interactions, underscores the need for an integrative approach that transcends traditional psychopathological frameworks. While substantial progress has been made in delineating its systemic complications and molecular mechanisms, translation into effective and durable treatments has lagged behind. Current therapeutic strategies, though essential, often fall short due to high relapse rates and limited pharmacological efficacy. Moving forward, research should prioritize the identification of novel biological targets, the refinement of multimodal interventions, and the personalization of treatment according to the individual patient’s clinical and biological profile. Importantly, bridging basic science discoveries with clinical practice will be critical to improving outcomes and reducing the burden of this devastating disorder.

## Figures and Tables

**Table 1:** Major studies from the past three years on medical nutrition management in patients with anorexia nervosa.

Author, Year	Type of Study	Findings	Remarks
Brennan et al., 2025 [184]	Semi-structured interviews were undertaken by 11 clinicians who had been working with adolescents aged 12–18 y.o. with anorexia nervosa (AN) who had been on a daily program.	The role of the dietician was pointed out as a crucial component of this intervention daily program.	The personalized nature of each nutritional interventionist turned out to be the most important determinant between the different phases of the nutritional program, especially between the weight restoration and maintenance phase.
Bohon et al., 2025 [185]	The authors have summarized key recommendations for AN management in the United States.	Early recognition of restrictive AN in the population together with family interventions and the help from a multidisciplinary team is of utmost importance.	The importance of screening tools for eating disorders in the general population has been stressed in this review manuscript.
Dozio et al., 2025 [186]	Observational study involving 79 patients with AN in Italy who had been admitted for management of AN.	The results after 6 months of medical management of these patients supported that early fat and protein restoration in these patients together with their close monitoring were very helpful.	The authors concluded that sustained medical nutritionists care and the implementation of a multidisciplinary team for as long as 6 months revealed encouraging results.
Brennan et al., 2024 [187]	A retrospective cohort study involving 92 patients with AN from Maudsley Center for Child and Adolescent Eating Disorders in London, United Kingdom.	The results of this study supported that there was no difference regarding weight gain among patients who received dietician’s guidance compared to those who did not.	The authors concluded that more information is needed in order to timely intervene with the consultation of a registered dietician among patients with AN.
Ayrolles et al., 2024 [188]	A scoping review from France regarding patients with early-onset pre-pubertal AN.	The authors have pointed out that there is lack of information regarding this particular group of early-onset AN.	The authors concluded that much more research is mandatory in this early-onset pre-pubertal group of patients with AN to better characterize and manage them.
Teo et al., 2024 [189]	A retrospective cohort study involving 47 patients with AN in Singapore.	Patients with AN can achieve weight restoration with a more dynamic management and for less duration of in-hospital stay with safety.	The authors concluded that in Asia and from their patient cohort, a shorter and more dynamic calorie administration could result in a lower duration of in-hospital stay. However, they have highlighted that the incidence of the refeeding syndrome should be further studied in this Asian group of patients.
Campos del Portillo et al., 2024 [190]	A consensus report from Spain.	The authors reported that outpatient management was most often preferred. However, there should be frequent re-assessment even at out of hospital care of these patients by a multidisciplinary team.	The authors point out the significance of alertness for the refeeding syndrome, especially among very malnourished patients with AN.
Hunter et al., 2024 [191]	A qualitative study interviewing 14 female patients with AN in the UK to evaluate the effects of a plant-based diet.	The authors proposed that a plant-based diet could be motivating for patients with AN.	The authors concluded that the participants found this plant-based diet motivating and that it was giving them good support for healthier eating habits.
Cinelli et al., 2023 [192]	An observational study including 8 outpatients with AN in Rome, Italy.	The patients were seen at baseline and after 6 months. The Mediterranean Diet was reported to be a good choice regarding weight management among these patients with AN.	The authors concluded that the Mediterranean Diet could offer a very promising dietary plan in patients with AN. However, the group consisted of only 8 patients.
Sthener et al., 2023 [193]	An editorial manuscript.	The authors reported that the initiation of nutritional management should be gradually increased during the first days due to the increased risk of refeeding syndrome.	The authors pointed out that the use of parenteral nutrition should be restricted among patients with AN due to increased risk for infections and metabolic adverse effects.

## Data Availability

Not applicable.

## Abbreviations

5-HT: serotonin; ACT: acceptance and commitment therapy; ACTH: adrenocorticotropic hormone; ADH: antidiuretic hormone; ALT: alanine aminotransferase; AN: anorexia nervosa; AST: aspartate aminotransferase; BDNF: brain-derived neurotrophic factor; BMD: bone mineral density; BMI: body mass index; CBC: complete blood count; CD: Crohn’s disease; CBT: cognitive behavioral therapy; CeD: celiac disease; CKD: chronic kidney disease; CKD-EPI: Chronic Kidney Disease Epidemiology Collaboration; CNS: central nervous system; COX: cyclooxygenase; CRH: corticotropin-releasing hormone; DEXA: dual-energy X-ray absorptiometry; DBI: diazepam-binding inhibitor; ED: eating disorder; eGFR: estimated glomerular filtration rate; ESRD: end-stage renal disease; FGF21: fibroblast growth factor 21; FHH: functional hypogonadotropic hypogonadism; fT3: free triiodothyronine; fT4: free thyroxine; FSH: follicle-stimulating hormone; FMT: fecal microbiota transplantation; G-CSF: granulocyte colony-stimulating factor; GDF15: growth differentiation factor 15; GBA: gut–brain axis; GH: growth hormone; GHSR: growth hormone secretagogue receptor; GHSR1a: growth hormone secretagogue receptor 1a; GnRH: gonadotropin-releasing hormone; HIF-1α: hypoxia-inducible factor 1-alpha; HPA: hypothalamic–pituitary–adrenal; HPG: hypothalamic-pituitary-gonadal; HPT: hypothalamic–pituitary–thyroid; HF: heart failure; HLA: human leukocyte antigen; HHV-6: human herpesvirus 6; HHV-7: human herpesvirus 7; IBD: inflammatory bowel disease; IN-OT: intranasal oxytocin; INR: international normalized ratio; IR: insulin resistance; iTBS: intermittent theta-burst stimulation; IGF-1: insulin-like growth factor 1; LCN2: lipocalin-2; LEAP2: liver-expressed antimicrobial peptide 2; LH: luteinizing hormone; LPS: lipopolysaccharide; MACE: major adverse cardiovascular event; MASLD: metabolic dysfunction-associated steatotic liver disease; MIC-1: macrophage inhibitory cytokine-1; MRI: magnetic resonance imaging; MMP-8: matrix metalloproteinase-8; NMDA: N-methyl-D-aspartate; NK: natural killer; NLR: neutrophil-to-lymphocyte ratio; NLRP3: NLR family pyrin domain-containing protein 3; NTIS: non-thyroidal illness syndrome; PGE2: prostaglandin E2; PDW: platelet distribution width; PPHS: posterior pituitary high signal; PNS: peripheral nervous system; PUFAs: polyunsaturated fatty acids; QoL: quality of life; RCT: randomized controlled trial; rT3: reverse triiodothyronine; SCFAs: short-chain fatty acids; DSM-5: Diagnostic and Statistical Manual of Mental Disorders, Fifth Edition; T2D: type 2 diabetes; T3: triiodothyronine; TGF-β: transforming growth factor-beta; TLR-2: toll-like receptor 2; TLR-4: toll-like receptor 4; TNF-α: tumor necrosis factor-alpha; TRH: thyrotropin-releasing hormone; TSH: thyroid-stimulating hormone; UC: ulcerative colitis; VTE: venous thromboembolism; WFSBP: World Federation of Societies of Biological Psychiatry.
